# Characterization of Actinobacterial Communities from Arauca River Sediments (Colombia) Reveals Antimicrobial Potential Presented in Low Abundant Isolates

**DOI:** 10.2174/1874285801812010181

**Published:** 2018-05-31

**Authors:** Carolina Arango, Alejandro Acosta-Gonzalez, Claudia M. Parra-Giraldo, Zilpa A. Sánchez-Quitian, Russell Kerr, Luis E. Díaz

**Affiliations:** 1 Universidad de La Sabana, Campus del Puente del Común, Km 7 Autopista Norte de Bogotá, Chia, Colombia; 2 Unidad de Investigación en Proteómica y Micosis Humanas, Pontificia Universidad Javeriana, Carrera 7 No. 40-62, Bogotá, Colombia; 3 University of Prince Edward Island, 550 University Avenue, Charlottetown, PE C1A 4P3, Canada

**Keywords:** Actinobacteria, Antimicrobial, Bioprospecting, MALDI-TOF MS, Physicochemical pretreatments, Taxonomic dereplication

## Abstract

**Introduction:**

New strategies have been arisen to set a rapid and effective screening for selection of microorganism with bioactive potential. This study suggests that combination of physicochemical pretreatments and taxonomic dereplication of microbial collections through MALDI-TOF MS, facilitates the detection of low abundance actinobacteria with potential as a source of antimicrobial agents.

**Material and Methods:**

An unstudied microbial community from a tropical river sediment in Colombian Orinoquía is described, applying an extended cultivation strategy using physicochemical pretreatments, biological screenings and taxonomic dereplication through MALDI-TOF MS approach.

**Results:**

Actinobacteria-like isolates (790) were growth and their antimicrobial activity was assessed against methicillin-resistant *Staphylococcus aureus*, Vancomycin-resistant *Enterococcus faecium*, extended-spectrum β-lactamase *Klebsiella pnumoniae*, and clinical isolates of *Cladosporium cladosporioides* and *Epicoccum nigrum.* Seventy-eight isolates, belonging to the Streptomycetaceae family according to 16S rDNA analysis were found to have antimicrobial activity and were categorized as low abundance actinobacteria by MALDI-TOF MS.

**Conclusion:**

The results suggest that combination of physicochemical pretreatments and taxonomic dereplication of microbial collections through MALDI-TOF MS, facilitates the detection of low abundance actinobacteria with potential as a source of antimicrobial agents.

## 1 INTRODUCTION

The continued and poorly controlled use of antimicrobial compounds has led to the emergence of resistant strains which have increased since the early 1960's [1]. Well-known examples of resistant bacterial strains are Methicillin-resistant *Staphylococcus aureus* (MRSA), Vancomycin-resistant *Enterococcus faecium* (VRE) and *Klebsiella pneumoniae,* which are important nosocomial pathogenic bacteria responsible for a wide range of pathologies [2-4]. One of the primary goals in bioprospecting studies with microorganisms is to enhance natural product discovery which can be used in pharmaceutical or other industry sectors [5-9]. Actinobacteria are a group of Gram positive bacteria which have proven to be a prolific source of natural products, being *Streptomyces* its representative genus [5]. Although actinobacteria have historically been isolated from soil habitats, nowdays they are understood to be widely distributed in varied aquatic environments as well [16-18, 21-24], leading to the discovery of novel bioactive compounds [6, 20]. However, since the late 1980's, the rate of discovery of new natural products from microorganisms has declined [10] thus requiring the development of new research strategies that can detect strains with a broad biological potential. Such strategies could involve the stablishing of an accurate low-cost workflow using: sampling from unstudied environments, combination of sample processing techniques, biological screening and high throughput taxonomic dereplication methods [11-14]. Firstly, exploration and sampling of understudied microbial habitats, where physicochemical characteristics may confer specialized metabolic processes to microorganisms, could lead to the discovery of new bioactive metabolites [1]. Secondly, the use of physicochemical pretreatments and selective microbiological media for actinobacteria isolation could facilitate the selective culturing of actinobacterial strains that could be difficult to recover by other traditional methods [15]. And finally, the use of biological screening procedures and high throughput dereplication methods in large microbial collections could decrease analysis times, costs and associated experimental work [12, 14].

In Colombia, hydrographic system of the Orinoquía basin has numerous reports about biodiversity in animals and vegetation, however little is known about its microbial biodiversity [25, 26] suggesting that tropical rivers from this habitat can be considered as an unexplored source of actinobacteria with bioactive potential. Strategies to isolate actinobacterial microorganisms from environmental samples are related to the use of physicochemical pretreatments [17, 27-35], facilitating its observation while removing unwanted microorganisms [15, 36]. Screening of microbial collections from environmental samples is a time-consuming and costly process. However, workflow using biological screening and dereplication strategies, have been arisen as a new tool for natural products research [14]. Antimicrobial screening through direct confrontation methods and taxonomic dereplication through microbial proteomic fingerprints using Matrix-assisted laser desorption/ionization mass spectrometry (MALDI-TOF MS) [12, 37-40] are simple procedures to detect and select strains with bioactive potential that can be studied in further tests, minimizing workflow times and cost [14, 41].

The study described herein was designed to test if the combination of the above-described strategies (sampling from unstudied environments, culture-dependent extended techniques, biological screening and high throughput taxonomic dereplication) will lead to the detection of actinobacterial related isolates capable of producing antimicrobial metabolites.

## 2 MATERIALS AND METHODS

### 2.1 Sampling

Two zones of the Arauca river banks in Colombia were identified. Zone 1 was located 6 kilometers west of Arauca City within the Guafitas sector. Zone 2 was located in La Lorena sector, 17 kilometers east of Arauca City. In each zone, sampling was conducted in two nearby sites. Coordinates of sampling points in Zone 1 (Guafitas): 7 ° 04'21.5 “N 70 ° 49'38.7” W and 7 ° 04'22.4 “N 70 ° 49'49.7” W and, in Zone 2 (La Lorena) 7 ° 03'51.9 “N 70 ° 36'35.3” W and 7 ° 03'55.4 “N 70 ° 36'39.3” W (According to Resolution No. 1043/2015 Universidad de La Sabana). Sampling was conducted using a methacrylate tube (25 cm long and 5 cm in diameter) which was submerged and then extracted with sediment samples. All samples were transported to Universidad de La Sabana, preserving a replica of the collected material under refrigeration (4^o^ C) [42, 43].

### 2.2 Physicochemical Pretreatments, Plating and Cryopreservation of Isolates

For selective isolation of actinobacteria, five physicochemical pretreatments were chosen: calcium carbonate (C), phenol (F), thermal (T), microwave (M), sonication (U) and no pretreatment or direct seeding (S) as a control. A total of 5 g of each sediment sample was placed in a 50 mL sterile tube for each of the pretreatments and were used for serial dilutions (10 times fold dilutions). Subsequently, 100 µL of each dilution was seeded on humic acid agar with vitamins (HVA) [44, 45] and oatmeal agar (ISP3), with 50 mg/L nalidixic acid (Sigma-Aldrich^®^) and 100 mg of cycloheximide (Sigma-Aldrich^®^). Seeding was performed in duplicate and all petri dishes seeded were incubated at 30^o^C for 7 days in an Innova^®^ 42 Incubator.

For each seeded plate, actinobacteria isolation was conducted according to the morphology of the colonies (including their thin, flat, radial and possibly spore morphology). In total 790 actinobacteria-like isolates were obtained and kept in ISP3 agar plates. After 7 days of incubation at 30^o^ C and detected presence of aerial mycelium growth in ISP3 agar, one photographic record of aerial and substrate mycelium growth from each isolate was taken, using a Canon PowerShot ELPH 180 (20.0 MP) digital photographic camera. Each isolate was cryopreserved in glycerol solution 40% (w/v) and preserved at -70° C in a Revco™ High-Performance lab freezer (Thermo Scientific™) [29], under numerical coding, according to the sampling site and physicochemical pretreatment.

A brief description of each pretreatment is presented below: Calcium Carbonate Pretreatment (C): The protocol used was taken from Otoguro [10] with modifications: 5 mL of sterile saline solution (0.85 M) was added to a 10 mL sterile tube with 5 g of sediment sample and mixed with 0.5 g of calcium carbonate powder (Panreac^®^) using a Labnet^®^ vortex mixer. The resulting mixture was kept at room temperature (18^o^ C) for 1 hour. Phenol Pretreatment (F): A modification of the method described by Hayakawa [35] was followed: 5 g of the collected samples was mixed in 60 mL of phosphate buffer (5 mM at pH 7) with phenol (Sigma-Aldrich^®^) at 1.5% (w/v). The solution was agitated with a Labnet^®^ vortex mixer for 5 min at 300 rpm and then kept in an Innova^®^ 42 Incubator at 30^o^ C for 30 min, without agitation. Thermal Pretreatment (T): A modification of a protocol described by Niyomvong [29] was followed: 5 mL of NaCl sterile saline solution (0.85 M) was added to a 50 mL sterile tube with 5 g of sediment sample. The sample suspension was agitated with Labnet^®^ vortex mixer for 5 min at 300 rpm and then heated at 120^o^ C for 1 hour in an Innova^®^ 42 Incubator. Microwave Pretreatment (M): A modification of a protocol described by Wang [46] was followed. 5 mL of a sterile saline solution (0.85 M NaCl) was added to a 50 mL sterile tube containing 5 g of a sediment sample. The mixture was agitated using Labnet^®^ vortex mixer for 5 min at 300 rpm and then placed in a glass centrifuge tube which was placed in a beaker with 900 mL of water to reduce overheating. The beaker with the tubes was placed in a 2450 MHz Samsung^®^ microwave and irradiated at a power of 100 W for 45 seconds (single continuous pulse). Sonication Pretreatment (U): A modification of the method described by Qiu [32] was followed. 5 mL of a sterile saline solution (0.85 M NaCl) was added to a 50 mL sterile tube with 5 g of sediment sample. The mixture was taken to a Labnet^®^ vortex mixer for 5 min at 300 rpm and then placed in a glass centrifuge tube. Glass centrifuge tubes with samples were placed in an Elma^®^ Transsonic TI-H-10 water bath sonicator with 10% of power for 2 min. at 30° C. Direct Seeding (S): 5 mL of a sterile saline solution (0.85 M NaCl) was added to a 50 mL sterile tube with 5 g of a sediment sample. The mixture was agitated using Labnet^®^ vortex mixer for 5 min at 300 rpm.

### 2.3 Protein Profile Similarity Assessment by MALDI-TOF MS

To obtain protein profiles, each actinobacterial isolate was seeded in oatmeal agar-ISP3 and incubated for 48 hours at 30^O^ C. An inoculum of each pure culture was deposited in a well of a metal plate for analysis (Bruker Daltonics^®^) and 1μL of matrix was added (2.5 mg/mL of α-cyano-4-hydroxycinnamic acid HCCA Bruker Daltonics^®^) in 50% acetonitrile, 2.5% trifluoroacetic acid and 47.5% HPLC water-Sigma^®^). The mixture was allowed to dry at room temperature. The spectra for each isolate were obtained after 240 laser shots in six different regions within the well by a Microflex spectrometer LT MALDI-TOF MS (Bruker Daltonics^®^) and analyzed using Bruker Flex software and MALDI Biotyper RTC 3.0 (Bruker Daltonics^®^). The BTS standard (Bacterial Test Standard) *Escherichia coli* DH5 from alpha peptide was used as a calibration standard. All the spectra were analyzed from 2,000 to 20,000 Da and then compared with BDAL database provided by Bruker. All protein profiles were used to make a correlation dendrogram using RTC Biotyper 3.0 software.

### 2.4 Mass Spectra Dendogram Generation

A dendrogram was generated using the MALDI-TOF Biotyper 3.1 software. The mass spectra of each duplicate of each isolate were obtained. In the cases where the result did not lead to a reliable identification, the spectrum with the most resolution was considered for dendrogram generation. For dendrogram analysis, a protein profile of Bacterial Test Standard (BTS) from *Escherichia coli* (DH5α) (Bruker Daltonik GmbH, Bremen, Germany) was used as calibration tool and spectra of all isolates tested were analyzed as a core-oriented dendrogram using an arbitrary distance level of 700 as the cutoff.

### 2.5 Assessment of Antibacterial Activity

Cross-streak is a simple and rapid antibacterial method for biological screening in large microbial collections when the goal is to detect strains capable of inhibiting target bacteria growth [36]. In this case and following a protocol described by Undabarrena [16], each actinobacterial isolate was inoculated on a quarter of a petri dish with Muller-Hinton agar (MH-Oxoid^®^) in triplicate and was incubated in an Innova^®^ 42 Incubator at 30^o^C for 7 days, providing to each actinobacteria-like isolate enough time to avoid nutrient depletion in the media and produce compounds with antibacterial activity that will diffuse into the agar medium and that can be responsible for growth inhibition in pathogenic bacteria [36]. After incubation, four pathogenic bacteria (extended-spectrum β-lactamases *Klebsiella pneumoniae* (ATCC^®^ 700603^TM^), *Staphylococcus aureus*-MRSA (ATCC^®^ BAA¬44^TM^), *Enterococcus faecium* VRE-(ATCC^®^ 700221^TM^) and *Bacillus subtilis* (ATCC^®^ 21556 ^TM^)) were seeded in 4 cm perpendicular lines. Petri dishes were incubated in an Innova^®^ 42 Incubator at 37^o^ C for 24 h and bacterial growth against the actinobacterial isolate seeded, was observed. Antibiosis was indicated by visually observable growth inhibition on target organisms and results were recorded as follows: inhibition zone > 2 cm: +++ (good activity), inhibition zone between 1-2 cm: ++ (moderate activity), inhibition zone < 1 mm: + (weak activity) and – (no activity) [47]. Negative and positive controls using rifampicin, vancomycin, ciprofloxacin and nalidixic acid (50mg/mL) (Sigma-Aldrich^®^) for pathogenic bacteria were assessed in a petri dish with Muller-Hinton agar (MH-Oxoid^®^) and were incubated in an Innova^®^ 42 Incubator at 37^o^C for 24h. Actinobacterial isolates that produced a partial or complete inhibition of at least one of pathogenic bacteria were taken for subsequent antifungal assays.

### 2.6 Assessment of Antifungal Activity

Three fungal clinical isolates were used: two isolates of *Cladosporium cladosporioides* (clinical isolate C1 and clinical isolate C2) and one isolate of *Epicoccum nigrum* (clinical isolate C3). For each fungus, a fungal spore suspension was made from 7 days old cultures in Potato Dextrose Agar (PDA-Oxoid^®^) that were incubated previously in an Innova^®^ 42 Incubator at 25^o^ C. Briefly, colonies were covered with 5 mL of a sterile saline solution (0.85 M NaCl) and the surface was scraped using a sterile loop. The concentration of spores of each fungus in the solution obtained was quantified in a Neubauer chamber and adjusted to a final concentration of 0.5 - 2.0 x 10^5^ spore mL^-1^ [48]. Each inoculum was added to a 12-well microplate with 3 mL of PDA (Oxoid^®^) per well. Once PDA was solidified with fungal inoculum, a 3mm^2^ diameter plug from the actinobacteria isolate with antibacterial activity previously established, was placed on each well and incubated at 25^o^ C for 7 days, using terbinafine hydrochloride 1% (Sigma-Aldrich^®^) and isoconazole nitrate 1% (Sigma-Aldrich^®^) as positive controls. Following incubation, the inhibition halos produced by the actinobacterial plug against the fungi were measured. Inhibition zones were recorded as follows: diameter > 6 mm: +++ (good activity), diameter 2-6 mm: ++ (moderate activity), diameter < 2 mm: + (weak activity) and – (no activity) [47].

### 2.7 PCR Amplification and 16S rRNA Sequencing

Only actinobacterial isolates with antimicrobial activity (antibacterial and antifungal) were taken for further molecular identification, following protocols described by Forner [49]. Strains were grown in ISP2 at 30° C in an Innova^®^ 42 Incubator under continuous agitation (250 rpm) for 8 days. The cultures were then centrifuged in a Hettich Universal 320R centrifuge at 12,000 rpm for 10 min. for subsequent DNA extraction, as described elsewhere [48]. Primers 27F (5 'AGT TTG ATC CTG GCT CAG 3') and 1492R (5 'ACG GCT ACC TTG TTA CGA CTT 3') were used for 16S rDNA amplification using a thermocycler (iCycler BioRad^®^) with the following conditions: initial denaturation at 95° C for 3 min., 35 cycles of 95 for 45 sec, 54° C for 1 min., 72^o^ C for 1 min., followed by a final extension at 72° C for 5 min. Amplification was confirmed by gel electrophoresis with 1.5% agarose with SYBR Green and molecular weight marker Cat Mwlon 300 (Invitrogen^®^). Sequences were assembled using the Chromas and BioEdit Sequence Alignment Editor version 7.2.5 software and compared with SILVA Living Tree Project database.

## 3 RESULTS

### 3.1 Physicochemical Pretreatments Effects on Isolation of Actinobacteria-like Species

The sampling of river bank sediment was followed by the recording of physicochemical characteristics at each location. The selected locations on the Arauca river were found to be strongly acidic [50] with low organic matter and low micronutrients (Ca^2+^, K^+^, Mg^2+^ and Na^+^) (Table **1**).

Five physicochemical pretreatments based on literature precedents were applied the river bank sediments of the Arauca River (Colombia) with the goal of selectively isolate as many actinobacterial strains as possible. The pretreatments here chosen were: calcium carbonate (C), phenol (F), thermal or heat (T), microwave (M) and sonication (U). Additionally, a direct seeding procedure (S) was performed as a control. A total of 790 actinobacteria-like isolates were recovered. Collection points 1 and 2 located in zone 1 (Guafitas) gave the greater percentage of isolates (57%). Regarding the five sets of pre-treatments, the highest percentage of isolates were obtained using the microwave (20.9%) and the phenol (20%) pretreatment, followed by use of calcium carbonate (18.2%) and sonication (18.1%) (Table **2**).

### 3.2 Antimicrobial Activity of Isolates

Direct confrontation tests to detect antibacterial and antifungal activity against selected pathogens were conducted with all isolates (790). Seventy-eight strains (9.87% of total) showed both antibacterial and antifungal activity against at least one bacterium and one fungus tested Table **3**. Of this group, 42 isolates (53.84%) inhibited the growth of *Staphylococcus aureus* - MRSA, 21 (26.92%) inhibited the growth of *Enterococcus faecium* - VRE, 19 (24.35%) inhibited the growth of *Klebsiella pneumoniae* and 73 isolates (93.6%) inhibited the growth of *Bacillus subtilis*. With respect to antifungal activity, 63 (80.76%) isolates inhibited growth of clinical isolates of *Cladosporium cladosporioides* (C1), 64 (82.05%) of *Cladosporium cladosporioides* (C2) and 77 (97.46%) of *Epicoccum nigrum* (C3).

### 3.3 MALDI-TOF MS Analysis and 16S rRNA Identification

Parallel with the bioactivity screening, an exploratory strategy for microbial taxonomic dereplication of the 790 actinobacteria-like isolates using MALDI-TOF MS was conducted and analyzed through MALDI-TOF Biotyper 3.1 software. The distance level cutoff for dendrogram construction was set according to literature precedent. Distance level cutoffs of 150 to 850 have been used to construct dendrograms for differentiating species inside one genus [51, 52], indicating that the distance level in which species are identified varies significantly [12]. It is therefore difficult to define a strict distance level cutoff value for a large collection of samples without any other information [12, 39]. However, it is generally acceptable to work with major distance values [53] to group major clades in large biological collections and rapidly reveal unique isolates [12]. In our case, a dendrogram was generated using a distance level cutoff of 700, 91 clades were identified with four “major clades” grouping together (representing approximately 66,7% of total isolates; clade No. 90 with 345 individuals, clade No. 89 with 86 isolates, clade No. 87 with 48 strains and clade No. 91 with 48 isolates). Other clades grouped an average of 1 to 31 isolates and are referred to as “minority clades”. Of the 91 clades obtained, only twenty different clades included at least one isolate that exhibited bioactivity in our assays and were named as “bioactivity clades”. The 85% of these bioactivity clades belong to the previous called minority clades giving 57 bioactive strains (73.08%) from the total of 78 bioactive isolates Table **4**. These “bioactive isolates” were sent for further molecular identification by 16S rRNA sequencing where it was found that they belong to the Streptomycetaceae family (Table **5**).

## 4 DISCUSSION

As is evident from Table **2**, the use of microwave, phenol, calcium carbonate and sonication pretreatments each gave rise to similar numbers of isolates (ca. 20%). The low number of isolates resulting from thermal pretreatment (2.6%) indicated the presence of microbes with low resistance to temperatures above 100^o^C for long periods [19]. The isolates demonstrating activity against at least one microbe were derived mainly from the phenol, sonication and microwave pretreatments Table **2**. It has been established that phenol can cause a leakage of cellular contents by first injuring lipid-containing membranes and then producing hydrogen bonding with vital proteins such as microbial enzymes [54]. On the other hand, bubbles formed through the cavitation process during sonication pretreatments can generate pressure which leads to breaking of the cell wall in microorganisms [55]. Finally, molecules such as water, DNA and proteins, respond to non-ionizing waves (microwaves) producing friction with neighboring molecules resulting in vibration and cellular damage [27]. Although these three physicochemical pretreatments have been used previously, they serve as a powerful tool for actinobacteria isolation due to their capacity to inhibit the growth of other taxa of microorganisms without producing lethal damage to actinobacteria [54]. It is possible that resistance to such treatments could be associated with a singular feature of these actinobacteria making them capable of producing different responses against an array of harsh condition. However, it should be noted that there is little experimental verification of such associations [46]. Importantly, the treatments used in this study can serve as efficient methods to isolate actinobacteria with different metabolic characteristics [35, 46]. The use of calcium carbonate proved to be ineffective as a method to isolate actinobacteria as this treatment yielded similar results to that from untreated samples. Our data can be rationalized by the fact that actinobacteria inhabiting these river bank environments can generate tolerance to changes of pH while using calcium carbonate. Previous reports have found that *Streptomyces* belonging to acid soils behave as acid-tolerant bacteria due to the different concentration of exchangeable hydrogen present in the sample [56]. Arauca sediments have been reported to have a pH of 4.0 - 5.0, characteristic that was also found in our data Table **1** and seen in Colombian Orinoquia and Amazonia basins. Meanwhile, true acidophilic actinobacteria could not grow at pH values greater than 6.5, acidotolerants can be found in media with pH values up to 7.5 [57-59]. According to these pH ranges, microorganisms herein isolated can be classified as acidophilus or acidotolerants. Therefore, increasing pH levels in these moderately acidic sediment samples, using calcium carbonate, would favor isolation of acidotolerant actinobacteria.

While the use of high temperatures (120^o^C) for prolonged periods has been described as a pretreatment that favors isolation of different genera of actinobacteria with resistant spores to desiccation, in this study was not possible to obtain a high number of isolates Table **2**. Unlike other pretreatments, where at least nine bioactive isolates were detected, heat pretreatment only gave a single unique isolate (isolate 864). A combined effect between high temperatures and acidity levels in samples could explain these results. In acidophilic and acidotolerant bacteria, H^+^ uptake from the surrounding medium depends on membrane fluidity, maintaining H^+^ homeostasis. This membrane fluidity may be affected by changes in environmental temperature conditions, decaying at low temperatures preventing uptake H^+^ or increasing at high temperatures due to a rise in membrane permeability which ultimately results in a lethal acidification of cell cytoplasm [60]. It is possible that this latter effect, during thermal pretreatment used in this study, could cause a drastic decline in actinobacteria isolation due to lethal acidification.

Differences in the numbers of isolates with antimicrobial activities from the four sample points was observed. In this case, sampling point 1 gave rise to the highest number of bioactive isolates (44), followed by point 4 (19), while points 2and 3 only had 9 and 6 isolates respectively (Table **2**).

Points selected for sediment sampling on the Arauca river banks, were acidic [50] with low organic matter and low micronutrients (Ca^2+^, K^+^, Mg^2+^ and Na^+^) Table **1**. Relative humidity at points 1 and 4 was lower than at points 2 and 3 (Table **1**).

Taking into account that moisture content in soil reduces aeration and decreases O_2_ uptake [61], sediment samples points 2 and 3 can be considered as samples with low oxygen, compared with sediment of points 1 and 4. This difference could influence the amount of bioactive isolates found, indicating that a medium-low content of moisture in sediments of tropical rivers, favors isolation of actinobacterial strains with antimicrobial bioactivity.

Using MALDI-TOF MS as a screening tool for taxonomic dereplication for large actinobacteria collections through similarity protein profile grouping, 91 clades or groups were identified and twenty of them included at least one bioactive isolate Table **4**. These 20 clades were termed “bioactive clades” and, interestingly, were part of the “minority clades” or clades with a maximum of 31 isolates. Minority clades grouped actinobacteria isolates with similar protein profile but were difficult to isolate due to their slow growth during the isolation process. This result indicates that the isolation strategy used in this study, was an effective approach to isolate actinobacteria strains that produce antibacterial metabolites. 16S sequence analysis indicates that these isolates belong to the Streptomycetaceae family Table **5** which is not unexpected given that these bacteria are a prolific source of antimicrobial agents [62-66].

## CONCLUSION

We report the isolation of different *Streptomyces* strains belonging to minority groups of cultivable actinobacterial isolates, that produce metabolites with antibacterial activity against Methicillin-resistant *Staphylococcus aureus*-MRSA, Vancomycin-resistant *Enterococcus faecium*-VRE and extended-spectrum β-lactamase *Klebsiella pnumoniae* and with antifungal activity against clinical isolates of *Cladosporium cladosporioides* and *Epicoccum nigrum,* through the combination of different methodologies. This supports our initial hypothesis that understudied environments such as Colombian aquatic tropical environments are a valuable resource for bioprospecting. Further, the use of a combinatorial isolation strategy focused on freshwater environmental sampling, physicochemical pretreatments such as phenol, sonication and non-ionizing radiation, and finally high throughput taxonomic dereplication through MALDI-TOF MS technology, revealed that minority groups of cultivable actinobacterial isolates leads to the isolation of actinobacteria that are a promising resource for antimicrobial drug discovery.

## Figures and Tables

**Table 1:** Values of pH, percentage of organic matter, relative humidity and concentration of Ca^2+^, K^+^, Mg^2+^ and Na^+^ from Arauca river bank collection sites.

**Sample Point**	**pH**	**%OM**	**Ca** ^2+^ **(meq/100g)**	**K** ^+^ **(meq/100g)**	**Mg** ^2+^ **(meq/100g)**	**Na** ^+^ **(meq/100g)**	**Relative Humidity (%)**
P1	4,8	0,63	1,4	0,14	1,07	0,09	19,65
P2	4,2	0,58	3,6	0,09	0,96	0,04	23,49
P3	4,4	1,2	3,2	0,29	1,44	0,02	30,41
P4	4,9	1,27	4,2	0,09	2,88	0,04	21,97

**Table 2:** A number of strains were isolated from Arauca river sediment samples. The number in each column compares the total actinobacteria-like isolates obtained after the applied physicochemical pretreatment versus the total number of isolates with positive antimicrobial bioactivity. Physicochemical pretreatments are abbreviated as follows: U (Sonication); C (Calcium carbonate); M (Microwave); F (Phenol); T (Thermal) and S (Direct Seeding).

**Pretreatment**	**Sample Point**				**Total Isolates**
	**Point 1**	**Point 2**	**Point 3**	**Point 4**	
S	35/5	55/0	22/0	47/4	159/9
C	34/6	34/2	36/1	40/4	144/13
F	51/12	46/1	32/4	29/3	158/20
M	47/11	57/3	24/0	37/3	165/17
U	43/10	41/3	26/1	33/4	143/18
T	4/0	3/0	3/0	11/1	21/1
**Total Isolates**	214/44	236/9	143/6	197/19	790/78

**Table 3:** Bioactivity results of actinobacterial isolates with both antibacterial and antifungal activity against at least one bacterium and one fungus tested. Inhibition zones observed from the antibacterial assay were recorded as follows: > 2 cm: +++ (good activity), inhibition zone between 1-2 cm: ++ (moderate activity), inhibition zone < 1 mm: + (weak activity) and – (no activity). Inhibition zones observed from the antifungal assay were recorded as follows: diameter > 6 mm: +++ (good activity), diameter 2-6 mm: ++ (moderate activity), diameter < 2 mm: + (weak activity) and – (no activity).

**ISOLATE**	**MRSA**	**VRE**	***Klebsiella pneumoniae***	***Bacillus subtilis***	***Cladosporium cladosporioides C1***	***Cladosporium cladosporioides C2***	*Epicoccum nigrum C3*
**5**	+++	+	-	++	++	++	++
**67**	++	-	++	++	-	-	++
**93**	+	-	-	++	++	+++	+++
**104**	-	-	+	-	-	-	++
**112**	-	-	+	+	++	++	++
**126**	+++	+++	-	+++	++	++	+++
**135**	+	-	++	+	-	-	+
**138**	+	-	+++	++	-	-	++
**140**	-	-	-	+	-	-	+
**145**	+++	+++	+	+++	-	-	+++
**152**	+	+	-	++	+++	+++	+++
**160**	++	-	-	+++	++	++	++
**197**	-	-	-	++	+++	+++	+++
**201**	+	-	+++	+++	++	++	+++
**208**	+	+	+	++	++	++	++
**213**	-	-	-	+	-	-	+
**220**	-	-	-	++	-	-	++
**243**	++	-	-	-	-	++	++
**246**	+	-	++	+	-	-	++
**247**	-	+	-	++	++	++	++
**276**	+++	++	-	+++	++	++	+++
**288**	-	+	-	++	++	++	++
**290**	++	++	-	+	++	++	+++
**292**	+	+	-	++	+++	+++	+++
**297**	-	-	+	-	+	-	++
**326**	++	++	-	++	++	++	+++
**330**	-	-	-	++	+	+	++
**335**	-	+	-	-	++	++	++
**356**	-	-	-	++	-	-	++
**365**	+	-	-	+	+++	+++	+++
**376**	-	++	+	+	+++	++	+++
**377**	-	-	++	+++	++	++	++
**381**	++	-	-	++	-	++	++
**382**	-	-	-	+	-	-	+
**388**	-	-	-	+	++	++	+++
**397**	-	-	-	++	++	-	++
**439**	+	-	-	++	+++	+++	++
**443**	-	-	-	++	+++	++	+++
**444**	+	-	-	+	++	++	++
**445**	++	-	-	++	+++	++	+++
**448**	+	-	-	+	+++	+++	+++
**479**	-	-	-	+	+++	+++	+++
**481**	-	-	+	++	++	++	-
**515**	++	-	-	++	++	++	+
**516**	-	+	-	+	+++	+++	+++
**530**	-	-	-	+	+++	+++	+++
**531**	++	+	-	+	++	++	++
**559**	+	-	-	++	++	++	++
**568**	-	-	-	+	++	++	+++
**571**	+++	-	-	++	+++	+++	+++
**572**	+++	-	-	++	+++	++	+++
**575**	-	-	++	+++	++	++	++
**612**	+	-	-	+	+++	+++	++
**616**	+++	+	-	+++	++	++	++
**619**	+++	-	-	+++	+++	+++	+++
**626**	++	-	+	++	++	++	++
**627**	+++	+	-	++	+	+	++
**636**	-	+	-	+	++	+++	+++
**652**	++	-	-	++	+	+	++
**659**	-	-	-	+	+++	++	+++
**660**	++	-	-	++	+	+	++
**663**	+	-	-	+++	++	++	++
**689**	+	-	-	++	+++	++	+++
**691**	++	+	-	++	-	-	++
**694**	++	++	-	++	-	+++	+++
**723**	-	-	-	+	++	++	++
**736**	++	+	-	++	+++	+++	+++
**744**	++	-	-	++	++	++	++
**745**	-	-	-	++	++	++	++
**766**	-	-	-	++	+	+	++
**775**	-	-	-	+	+++	+++	++
**790**	+	-	+	+	+++	+++	++
**805**	-	-	++	-	++	++	++
**809**	-	-	++	++	++	++	++
**837**	-	-	-	+	++	++	+++
**840**	-	-	+	+	++	++	++
**847**	-	-	-	+	++	++	+++
**864**	-	-	-	+	+++	++	+++

**Table 4:** Clades obtained through MALDI-TOF MS with at least one bioactive isolate. Percentage of bioactive isolates in each clade is shown. Clades 89, 90 and 91 belonged to majority clades.

**CLADE**	**Total Isolates**	**Bioactive Isolates**	**% Bioactive Isolates**
9	5	4	80
20	31	1	3,2
22	1	1	100
42	1	1	100
43	2	2	100
50	1	1	100
54	3	2	66,7
55	7	4	57,1
57	6	6	100
58	5	1	20
59	19	9	42,1
60	27	21	77,7
61	1	1	100
68	4	1	25
75	11	1	9,1
78	4	3	75
79	4	1	25
89	86	2	2,3
90	345	13	3,7
91	48	3	6,3
**TOTAL**	**790**	**78**	**9,87**

**Table 5:** Clades obtained by MALDI-TOF MS with at least one actinobacterial isolate with antimicrobial activity. 16S rRNA molecular identification was obtained using SILVA Living Tree Project database. Sampling site and physicochemical pretreatment are included – U: Sonication; C: Calcium carbonate; M: Microwave; F: Phenol; T: Thermal; S: Direct Seeding.

**Clade**	**No. Isolates**	**No. Bioactive Isolates**	**Bioactive Isolate**	**Pretreatment**	**Sample Point**	**Identification (98,7%)**
9	5	4	439	U	P1	*Streptomyces hiroshimensis*
			559	C	P4	*Streptomyces lacticiproducens*
			736	C	P4	*Streptomyces hiroshimensis*
			775	M	P4	*Streptomyces hiroshimensis*
20	31	1	636	M	P2	*Nonomuraea candida*
22	1	1	388	M	P2	*Streptomyces werraensis*
42	1	1	694	U	P4	*Streptomyces* sp.
43	2	2	276	S	P4	*Streptomyces griseofuscus*
			290	C	P1	*Streptomyces albospinus*
50	1	1	444	U	P1	*Streptomyces lunalinnaresii*
54	3	2	652	M	P4	*Streptomyces* sp.
			515	S	P4	*Streptomyces sioyaensis*
55	5	4	376	M	P1	*Streptomyces luteireticuli*
			445	U	P1	*Streptomyces* sp.
			571	F	P1	*Streptomyces luteireticuli*
			572	F	P1	*Streptomyces luteireticuli*
57	6	6	288	C	P1	*Streptomyces sparsogenes*
			377	M	P1	*Streptomyces cinnamoneus*
			481	S	P1	*Streptomyces griseoaurantiacus*
			575	F	P1	*Streptomyces cinnamoneus*
			809	F	P1	*Streptomyces cinnamoneus*
			840	F	P1	*Streptomyces cinnamoneus*
58	5	1	104	C	P4	*Streptomyces albolongus*
59	19	9	247	S	P1	*Streptomyces* sp.
			397	M	P2	*Streptomyces morookaense*
			568	F	P1	*Streptomyces morookaense*
			612	M	P1	*Streptomyces luteosporeus*
			689	U	P4	*Streptomyces pseudoechinosporeus*
			745	F	P1	*Streptomyces morookaense*
			766	M	P1	*Streptomyces orinoci*
			837	C	P1	*Streptomyces lacticiproducens*
			864	T	P4	*Streptomyces lacticiproducens*
60	29	21	5	S	P1	*Streptomyces lunalinnaresii*
			135	F	P3	*Streptomyces aureocirculatus*
			138	F	P3	*Streptomyces puniceus*
			160	M	P1	*Streptomyces mobaraensis*
			197	M	P4	*Streptomyces misionensis*
			201	U	P1	*Streptomyces lunalinnaresii*
			208	U	P1	*Streptomyces kasugaensis*
			326	F	P1	*Streptomyces mashuensis*
			381	M	P1	*Streptomyces ramulosus*
			448	U	P2	*Streptomyces albulus*
			479	S	P1	*Streptomyces mashuensis*
			516	S	P4	*Streptomyces* sp.
			531	C	P1	*Streptomyces hiroshimensis*
			616	M	P1	*Streptomyces yunnanensis*
			619	M	P1	*Streptomyces yunnanensis*
			626	M	P1	*Streptomyces lunalinnaresii*
			627	M	P1	*Streptomyces lydicus*
			659	U	P1	*Streptomyces yunnanensis*
			660	U	P1	*Streptomyces mobaraensis*
			723	C	P1	*Streptomyces albospinus*
			847	U	P2	*Streptomyces albospinus*
61	1	1	213	U	P1	*Streptomyces lanatus*
68	4	1	530	S	P4	*Streptomyces noursei*
75	11	1	243	U	P4	*Streptomyces sparsogenes*
78	4	3	365	F	P4	*Streptomyces lydicus*
			744	F	P1	*Streptomyces hygroscopicus*
			790	U	P3	*Streptomyces iranensis*
79	4	1	805	C	P3	*Streptomyces sioyaensis*
89	86	2	330	F	P1	*Streptomyces puniciscabei*
			356	F	P4	*Streptomyces corchorusii*
90	345	13	67	C	P2	*Streptomyces puniciscabei*
			93	C	P4	*Streptomyces griseofuscus*
			112	F	P1	*Streptomyces lacticiproducens*
			126	F	P2	*Streptomyces padanus*
			152	F	P4	*Streptomyces albospinus*
			220	U	P2	*Streptomyces phaeoluteigriseus*
			292	C	P1	*Streptomyces murinus*
			297	C	P2	*Streptomyces lanatus*
			335	F	P1	*Streptomyces kebangsaanensis*
			382	M	P1	*Streptomyces puniciscabei*
			443	U	P1	*Streptomyces griseofuscus*
			663	U	P1	*Streptomyces cinnamoneus*
			691	U	P4	*Streptomyces* sp.
91	48	3	140	F	P3	*Streptomyces misionensis*
			145	F	P3	*Streptomyces parvulus*
			246	S	P1	*Streptomyces graminisoli*
